# Machine Learning-Based Prediction of Delayed Cerebral Ischemia and Functional Outcome in Aneurysmal Subarachnoid Hemorrhage: The Role of CNS Infection-Related Parameters

**DOI:** 10.3390/biomedicines14081853

**Published:** 2026-08-18

**Authors:** Vanessa Magdalena Swiatek, Tom Tobias Kummer, Conrad-Jakob Swiatek, Lea Ehrhardt, Klaus-Peter Stein, Ali Rashidi, Robert Werdehausen, I. Erol Sandalcioglu, Sylvia Saalfeld, Belal Neyazi

**Affiliations:** 1 Department of Neurosurgery, Otto-von-Guericke University, 39106 Magdeburg, Germany; vanessa.swiatek@med.ovgu.de (V.M.S.); conrad-jakob.swiatek@med.ovgu.de (C.-J.S.); erol.sandalcioglu@med.ovgu.de (I.E.S.); 2 Department of Anesthesiology and Intensive Care Medicine, Otto-von-Guericke University, 39106 Magdeburg, Germany; 3 Department of Data Analysis in Life Sciences, Ilmenau University of Technology, 98693 Ilmenau, Germany; lea.ehrhardt@tu-ilmenau.de; 4 Research Campus STIMULATE, 39106 Magdeburg, Germany; 5 Department of Medical Informatics, University Hospital Schleswig-Holstein Campus Kiel, 24105 Kiel, Germany

**Keywords:** aneurysmal subarachnoid hemorrhage, delayed cerebral ischemia prediction, functional outcome prediction, machine learning, aggregated longitudinal parameters

## Abstract

**Background/Objectives**: Aneurysmal subarachnoid hemorrhage (aSAH) carries high morbidity and mortality, with delayed cerebral ischemia (DCI) as a major secondary complication. Although infection has increasingly been implicated in DCI pathogenesis, the role of central nervous system (CNS) infection remains unclear. We evaluated CNS infection-related parameters for predicting DCI and functional outcome after aSAH using machine learning. **Methods**: This retrospective single-center study included 191 patients with confirmed aSAH treated in the intensive care unit between 2019 and 2024. Demographic, clinical, radiographic, treatment-related, microbiological, cerebrospinal fluid (CSF), and longitudinal laboratory data were extracted from electronic records. DCI was analyzed as a binary outcome, and discharge functional outcome was dichotomized using the modified Rankin Scale (0–2 vs. 3–6). Twelve feature selection methods and twelve classifiers were evaluated using five-fold cross-validation. **Results**: For DCI prediction, the models achieved a mean accuracy of 0.96, F1-score of 0.96, and AUC of 0.98. Key predictors included antibiotic therapy, aneurysm treatment modality, history of thrombosis, and EVD revision, along with mean albumin, red cell distribution width, INR, minimum aspartate aminotransferase, and CSF glucose, lactate, nucleated cell count, and cellular debris. For functional outcome prediction, accuracy was 0.948, F1-score 0.95, and AUC was 0.97. Predictors comprised cerebral vasospasm, number of spasmolysis procedures, pre-admission anticoagulation, CSF pathogen detection and type, aPTT, fibrinogen, aspartate aminotransferase, lactate dehydrogenase, and CSF mononuclear cell proportion, leukocyte count, and glucose concentration. **Conclusions**: Machine learning models integrating clinical, systemic, and CSF-derived parameters demonstrated excellent performance in predicting both DCI and functional outcome after aSAH. The identified predictors highlight the importance of the neurocritical care course, including infection-related and thromboinflammatory processes, and extend beyond traditional vasospasm-centered paradigms. These findings may inform risk stratification, although the temporal relationship between several predictors and outcome onset means the models should be regarded as hypothesis-generating rather than tools for early clinical prediction.

## 1. Introduction

Aneurysmal subarachnoid hemorrhage (aSAH) remains a devastating cerebrovascular emergency with substantial morbidity and mortality despite advances in neurocritical care and endovascular techniques. While early brain injury from the initial hemorrhagic insult contributes to poor outcomes, delayed cerebral ischemia (DCI) represents a major secondary complication that develops in approximately 20–30% of patients, typically between four and 14 days after aneurysm rupture [1,2,3]. DCI is a leading cause of death and disability among aSAH survivors, and its pathophysiology extends beyond the traditional concept of large vessel vasospasm (CVS) to encompass a complex interplay of microvascular dysfunction, neuroinflammation, cortical spreading depolarizations, and autoregulatory failure [4,5,6].

The complex pathophysiology of DCI has led to extensive research aimed at identifying potentially modifiable risk factors associated with its occurrence. Well-established predictors include initial clinical severity, reflected by the Hunt and Hess scale and the World Federation of Neurosurgical Societies score, as well as the radiographic extent of hemorrhage assessed by the modified Fisher scale. Additional factors linked to an increased risk of DCI comprise intraventricular hemorrhage, hydrocephalus, female sex, and pre-existing comorbidities such as arterial hypertension and diabetes mellitus [3,7]. However, emerging evidence suggests that systemic and intracranial infections play a previously underappreciated role in DCI pathogenesis. Nosocomial infections, including pneumonia, urinary tract infections, and central nervous system (CNS) infections, are highly prevalent in critically ill aSAH patients and have been independently associated with increased DCI risk and worse functional outcomes [7,8,9].

The mechanistic link between infection and DCI is hypothesized to involve amplification of systemic and neuroinflammatory cascades. After aSAH, hemoglobin degradation products and early brain injury initiate a pronounced neuroinflammatory cascade. This response is marked by activation of resident and peripheral immune cells, increased release of pro-inflammatory cytokines, and migration of monocytes and lymphocytes into the cerebrospinal fluid (CSF) and brain parenchyma [10,11,12,13]. Superimposed infection further exacerbates this inflammatory milieu, promoting microvascular thrombosis, endothelial dysfunction, and impaired cerebral autoregulation—processes that collectively increase susceptibility to ischemic injury [8,14]. Notably, in most cases, the diagnosis of infection temporally precedes the onset of DCI, supporting a potential causative relationship rather than mere association [8].

CNS infections, particularly ventriculitis and meningitis associated with external ventricular drainage (EVD), represent a specific subset of nosocomial complications in aSAH patients. The incidence of EVD-associated infection ranges from 0 to 22%, with risk factors including intraventricular hemorrhage, prolonged catheterization, catheter manipulation, and prior craniotomy [15]. Diagnosis of CNS infection in this population is particularly challenging, as blood contamination of the CSF and the systemic inflammatory response inherent to aSAH can mimic infectious processes, rendering conventional CSF parameters (leukocyte count, glucose, and protein) less specific [16,17]. Despite these diagnostic complexities, intracranial infection has been identified as an independent risk factor for DCI in meta-analytic studies, with odds ratios as high as 25.3 for ventriculitis [7,8].

The advent of machine learning techniques has revolutionized outcome prediction in aSAH by enabling integration of heterogeneous clinical, radiographic, and laboratory data to model complex, non-linear relationships. Recent studies have demonstrated that machine learning algorithms outperform conventional Logistic Regression in predicting DCI and functional outcomes, achieving area under the receiver operating characteristic curve (AUC) values ranging from 0.75 to 0.89 [18,19,20,21,22]. These models have incorporated diverse predictors such as clinical severity scores, radiographic features, computed tomography (CT) perfusion parameters, and peripheral inflammatory markers. However, to date, no machine learning study has specifically examined the influence of CNS infections or systematically integrated microbiological and cerebrospinal fluid parameters as predictors of DCI.

This study addresses this critical knowledge gap by applying a comprehensive machine learning approach to investigate the role of CNS infection-related parameters in the development of DCI and functional outcomes among patients with aSAH. Unlike prior machine learning studies that have focused primarily on clinical severity scores, radiographic features, and peripheral inflammatory markers, this investigation systematically integrates a broad spectrum of variables spanning demographic and baseline characteristics, cardiovascular risk factors and comorbidities, initial radiographic findings, aneurysm-specific features, treatment modalities and procedural complications, clinical severity scores, hydrocephalus and CVS management, microbiological and cerebrospinal fluid infection-related parameters, and longitudinal peripheral blood inflammatory, hematologic, metabolic, and coagulation markers collected throughout the intensive care unit stay. By incorporating CNS infection-related parameters alongside traditional clinical predictors within a machine learning framework, we aim to: (1) elucidate the independent contribution of CNS infection-related parameters to DCI risk and functional outcomes in the broader aSAH population; (2) identify the most informative predictors of DCI and discharge functional status using multiple feature selection strategies; and (3) develop predictive models that may support risk stratification of patients and inform targeted therapeutic strategies to mitigate secondary brain injury after aSAH.

## 2. Materials and Methods

### 2.1. Materials

This retrospective single-center investigation comprised 193 neurosurgical intensive care unit (ICU) patients diagnosed with aSAH who received treatment at the Department of Neurosurgery, Otto von Guericke University Magdeburg, between April 2019 and May 2024. The study protocol, including data acquisition and analytical procedures, was reviewed and approved by the Ethics Committee of the Medical Faculty of Otto von Guericke University (RENOVA 94/20; approved 22 June 2020).

Following application of the five predefined inclusion criteria, 191 patients diagnosed with aSAH were enrolled in the study. Two patients were excluded due to incomplete clinical and imaging data.

The dataset comprised demographic characteristics, including age and sex, as well as information on cardiovascular risk factors, the presence of malignant or autoimmune disorders, and pre-existing medication. Hemorrhage-related variables covered the specific subtype of intracranial bleeding and radiological findings, such as intraventricular hemorrhage, hydrocephalus, ischemic lesions at admission, and midline shift. Aneurysm-specific parameters included rupture status, anatomical location, the presence of multiple intracranial aneurysms, and morphological features such as aneurysmal blebs. Further variables addressed the modality and timing of aneurysm treatment, procedure-related complications, occlusion rates, and the development and management of hydrocephalus. Data were also collected on the incidence, diagnostic workup, and therapeutic management of CVS and DCI. Clinical severity at presentation was quantified using the Hunt and Hess scale, Fisher scale, Glasgow Coma Scale (GCS), and the World Federation of Neurosurgical Societies grading system. Functional outcome was assessed at discharge according to the modified Rankin Scale (mRS).

In this study, microbiological analyses focused on cerebrospinal fluid samples obtained from external ventricular drains (EVDs) and, in the later clinical course, from lumbar drains. The collected variables included detection of pathogens in the CSF, specification of the identified microorganism, and documentation of antibiotic treatment. In addition, microscopic examination covered the presence of bacteria, fungal hyphae, budding yeast, granulocytes, erythrocytes, lymphocytes, and cellular debris. Further parameters comprised information on pathogen identification, the need for replacement of the EVD, and the total duration of drain placement. Comprehensive laboratory data from peripheral blood samples were also recorded. These included complete and differential blood counts, as well as differential leukocyte subsets reported both as percentages and absolute values. Inflammatory and organ function markers comprised C-reactive protein, liver enzymes, bilirubin, lactate dehydrogenase, albumin, cholinesterase, pancreatic amylase, lipase, creatinine, urea, and estimated glomerular filtration rate (eGFR). Coagulation parameters included activated partial thromboplastin time (aPTT), fibrinogen, international normalized ratio, antithrombin, and prothrombin time. Electrolytes and metabolic parameters encompassed calcium, chloride, glucose, potassium, and sodium. Antibiotic therapy was administered according to the institutional antibiotic guideline. Empirical therapy for suspected pneumonia or urinary tract infection consisted of piperacillin/tazobactam, whereas suspected CNS infection was empirically treated with a combination of meropenem and vancomycin. Once a causative pathogen was identified, antibiotic regimens were subsequently adjusted according to antimicrobial susceptibility testing.

CSF laboratory analyses further included macroscopic assessment, leukocyte and erythrocyte counts, proportions of polymorphonuclear and mononuclear cells, total nucleated cell count, glucose, protein, and lactate levels. All variables were collected longitudinally over the entire ICU stay and were manually extracted from the electronic medical records. We included patients with confirmed aSAH by applying the following inclusion criteria:

1. Confirmation of aSAH by cranial CT or CSF analysis obtained through lumbar puncture;
2. Admission to and management in an ICU after aneurysm rupture;
3. Complete availability of clinical records and imaging data for analysis.

Because these parameters were aggregated as mean, minimum, or maximum values across the entire ICU stay rather than restricted to a prespecified baseline or a defined pre-DCI time window, the resulting models characterize associations between the overall neurocritical care course and outcome rather than providing prediction from a fixed, strictly pre-outcome time point.

Within the analyzed cohort, 54 patients harbored multiple intracranial aneurysms, whereas 137 patients had a single aneurysm. The study population consisted of 69.6% women and 30.4% men. The average age at the time of aneurysm rupture was 56.5 years; the majority of patients (79%) were younger than 70 years, while 21% were aged 70 years or older. Detailed demographic characteristics of the patients and aneurysm features are summarized in Table 1.

### 2.2. Definitions

CVS was evaluated on the basis of clinical presentation and a multimodal diagnostic workup comprising transcranial Doppler sonography, computed tomography angiography, and intra-arterial digital subtraction angiography. For the present analysis, CVS was recorded only when confirmed by angiographic imaging. Cases with Doppler findings suggestive of CVS but without angiographic verification were categorized as not having CVS. DCI was defined in accordance with the criteria published by Vergouwen et al. [23]. Inclusion required that the onset of hemorrhage could be retrospectively determined within a maximum interval of one week, based on documentation from emergency medical services and ambulance reports.

All patients with aSAH received a standardized prophylactic regimen aimed at reducing the risk of DCI. This protocol consisted of continuous nimodipine administration (oral or intravenous), maintenance of mean arterial pressure above 90 mmHg, goal-directed normovolemic fluid therapy guided by laboratory values, and ensuring sufficient oxygen supply.

In our cohort, CNS infection was evaluated in the context of aSAH and the presence of an EVD, where infection is typically characterized as ventriculitis or meningitis associated with CSF diversion. According to the Infectious Diseases Society of America, healthcare-associated ventriculitis and meningitis are defined as infection of the CSF in patients with a CSF drain and are commonly accompanied by new or worsening altered mental status, fever, and an elevated CSF leukocyte count; however, these features lack specificity in patients with aSAH and EVD [15]. Diagnostic assessment is particularly challenging in this population, as blood contamination of the CSF and the systemic inflammatory response related to the hemorrhage itself may mimic infectious processes. The Infectious Diseases Society of America therefore recommends that infection be suspected in patients with EVD who develop new-onset fever, neurological deterioration, or rising CSF leukocyte counts, while emphasizing that clinical and routine laboratory findings alone are insufficient and that microbiological confirmation is required for a definitive diagnosis [15]. Reported rates of EVD-associated infection vary widely, ranging from 0 to 22%, with established risk factors including intraventricular or SAH, catheter manipulation or irrigation, prior craniotomy, and prolonged catheter duration [15]. Although conventional CSF parameters such as leukocyte count, glucose, and protein provide supportive information, their interpretation remains limited due to hemorrhage-related inflammatory changes [16,17,24]. Consequently, positive CSF culture remains the diagnostic gold standard, even though empirical antimicrobial therapy is frequently initiated based on clinical suspicion given the substantial morbidity associated with untreated infection [17].

Given these diagnostic challenges and the limited specificity of combined criteria in the setting of aSAH, we deliberately refrained from applying a composite definition of CNS infection in the present study. Instead, all potentially relevant individual parameters and surrogate markers were systematically collected and analyzed separately, without defining infection based on a predefined constellation of findings. Accordingly, throughout this manuscript, the term “pathogen detection” refers strictly to a positive CSF culture, without requiring the concurrent clinical criteria recommended by the Infectious Diseases Society of America for a diagnosis of healthcare-associated ventriculitis or meningitis. This operational definition cannot distinguish true CNS infection from culture contamination, particularly given the predominance of cutaneous commensal organisms in our cohort. Statements regarding “CNS infection” elsewhere in this manuscript should therefore be interpreted as referring to CSF pathogen detection and related surrogate markers rather than to confirmed clinical infection.

### 2.3. Explanation of the Machine Learning Approach

Following data acquisition and preprocessing, all relevant clinical variables were extracted and organized using Microsoft Excel (Office 2016, Microsoft Corporation, Redmond, WA, USA; https://www.microsoft.com/excel, accessed on 2 February 2026). The dataset specifically comprised the laboratory and microbiological CSF parameters collected during ICU stay and included the following parameters:

1. Sex;
2. Age at diagnosis;
3. Presence of comorbidities/vascular risk factors: Hypertension, diabetes, hypercholesterolemia, peripheral arterial disease, heart diseases, prior stroke, nicotine abuse, alcohol abuse, history of thrombosis, intake of contraception, history of malignant diseases, obesity, and autoimmune disease;
4. Intake of anticoagulants or antiplatelet agents before treatment;
5. Findings in the initial computed tomography scan: Type of bleeding, localization of intracerebral hemorrhage if applicable, presence of intraventricular hemorrhage, and midline shift or ischemic stroke;
6. Aneurysm characteristics: Rupture status, multiplicity, localization, shape, and presence of a blep;
7. Clinical scores: Hunt and Hess scale, Fisher scale, GCS, and the World Federation of Neurosurgical Societies scale at admission;
8. Type of aneurysm repair: Previous treatments and modality, current treatment and modality;
  - Surgical group: Type of craniotomy, presence of intraoperative rupture, application of temporary clipping, application of ICG angiography and micro-Doppler, evacuation of a possible intracerebral hemorrhage, performance of decompressive hemicraniectomy, postoperative ischemic and hemorrhagic complications as well as the need for revision, and occlusion of the aneurysm;
  - Endovascular group: Type of endovascular approach, number of treatments, ischemic and hemorrhagic complications as well as the need for revision, and aneurysm occlusion;
9. Hydrocephalus: Presence of hydrocephalus, placement of an EVD or a ventriculoperitoneal shunt, and application of actilysis;
10. CVS: Detection of CVS, implantation of an intracranial pressure or brain tissue oxygenation monitoring device, need for interventional spasmolysis, and date of CVS occurrence after aSAH;
11. Beyond the traditional aneurysm- and aSAH-related parameters, we included the following parameters linked to CSF infection:
  - CSF infection-related parameters: Pathogen identification in the CSF, duration of EVD placement, need for insertion of a new drain following pathogen detection, type of identified microorganism, administration of antibiotic therapy, macroscopic appearance, leukocyte count, erythrocyte count, proportion of polymorphonuclear and mononuclear cells, total nucleated cell count, glucose, protein, lactate, and microscopic assessment for bacteria, fungal hyphae, granulocytes, erythrocytes, cellular debris, budding yeast, and lymphocytes;
  - ICU parameters: Leukocyte count, erythrocyte count, hemoglobin, hematocrit, mean corpuscular volume (MCV), mean corpuscular hemoglobin, mean corpuscular hemoglobin concentration, red cell distribution width (RDW), platelet count, large platelet ratio, mean platelet volume (MPV), differential leukocyte counts (neutrophils, immature granulocytes, eosinophils, basophils, lymphocytes, and monocytes) reported both as percentages and absolute values, C-reactive protein, alanine aminotransferase, aspartate aminotransferase, bilirubin, eGFR, gamma-glutamyl transferase, glutamate dehydrogenase, creatinine, urea, lactate dehydrogenase, albumin, alkaline phosphatase, pancreatic amylase, cholinesterase, lipase, aPTT, fibrinogen, international normalized ratio, antithrombin, prothrombin time (Quick), calcium, chloride, glucose, potassium, and sodium.

For machine learning purposes, the dataset underwent systematic restructuring. The strategy for aggregating longitudinal variables and handling missing data is described in detail in Section 2.4.

DCI was modeled as a binary classification target based on its association with the extracted features. Discharge functional outcome was likewise dichotomized using the mRS, with scores of 0–2 classified as favorable and scores of 3–6 as unfavorable.

Feature standardization was performed in Python (version 3.11, 2025) using z-score normalization, and the processed data were subsequently exported and stored in Microsoft Excel format. Correlation analysis was conducted within Python to eliminate highly collinear variables, defined as a Pearson correlation coefficient greater than 0.85 [25]. The dataset was then partitioned using five-fold cross-validation, resulting in five separate training and test splits. Feature selection and classification procedures were executed independently within each fold. Details of the cross-validation strategy, class-imbalance handling, hyperparameter selection, and the AUC confidence interval calculation are provided in Section 2.4.

Initially, twelve feature selection techniques were evaluated to identify the most informative predictors. To further reduce dimensionality, limit residual inter-feature correlation, and decrease computational complexity, the final number of selected features was restricted to ten. This threshold was determined after testing multiple alternative cutoffs. The applied feature reduction methods included ReliefF (RLF) [26], Analysis of Variance (ANOVA), Chi-squared (Chi2) test, Linear Regression (LIR), Logistic Regression (LOR), CART Regression (CARTR), CART Classification (CARTC), Random Forest Regression (RFR), Random Forest Classification (RFC) [27], Fisher scale (Fisher), Laplacian Score [28], and Gradient Boosting (RB) [26]. Dictionaries were generated to systematically organize training and test datasets, feature names, and feature indices. These methods have been widely described as suitable approaches for feature selection in comparable analytical settings [29,30,31].

The selection of a broad range of feature reduction techniques was also guided by clinical considerations. Systemic deterioration after aSAH may result from heterogeneous pathophysiological mechanisms, which differ in their statistical behavior. While some processes manifest as linear trends, others present as non-linear fluctuations in physiological parameters. Employing complementary feature selection strategies therefore increased the likelihood of identifying clinically meaningful predictors of DCI across diverse physiological domains without methodological bias.

For each feature selection approach, twelve classification algorithms were trained and evaluated. The implemented classifiers comprised Neural Networks (NNs), Decision Trees (DTREEs), Support Vector Machines (SVMs), k-Nearest Neighbors (KNNs), Random Forest (RF), Logistic Regression (LRC), Gaussian Naive Bayes (GNB) [27], eXtreme Gradient Boosting (XGB) [26], Stochastic Gradient Descent (SGD), Bernoulli Naive Bayes (BNB), Ensemble Bagged Trees (EBTs), and AdaBoost (ABC) [27].

### 2.4. Model Development and Validation Strategy

Missing data and longitudinal aggregation were handled as follows. Variables exceeding a 50% missing data threshold were excluded prior to analysis. Rather than imputing missing values for the remaining parameters, incomplete cases were removed on a per-fold basis during cross-validation. Continuous variables were summarized across the ICU stay by calculating their minimum, maximum, and mean values or, where appropriate, transformed into time-dependent metrics such as time to event. Mean, minimum, and maximum values were chosen as aggregation statistics because they are straightforward to compute, clinically interpretable, and capture complementary aspects of each parameter’s course (central tendency and the most extreme low or high value, which is often of particular clinical relevance for laboratory and CSF parameters); more complex repeated-measures representations (e.g., slopes, time-weighted averages) were not pursued given the substantial missingness and irregular sampling intervals of the underlying longitudinal data.

Model validation was performed using stratified five-fold cross-validation (StratifiedKFold), which preserves the proportion of outcome classes (DCI vs. no DCI; favorable vs. unfavorable mRS) in each training and test split. Prior to model training, class imbalance was additionally addressed using a combination of oversampling and undersampling (MATLAB, version R2025b, MathWorks, Natick, MA, USA) applied to the training data. Within each fold, all twelve feature selection methods were applied using only that fold’s training data, and for each resulting feature set, all twelve classifiers were trained on the same training data and evaluated on the corresponding held-out test data; accuracy, F1-score, and AUC were recorded for every feature selection–classifier combination. For each fold, the combination achieving the highest test-fold accuracy was retained as that fold’s best-performing model, and its prediction was used for the final performance evaluation; overall accuracy, F1-score, AUC, and confusion matrix values were then calculated by pooling the best-performing predictions across all five folds.

Hyperparameters for all feature selection and classification algorithms were set to their default scikit-learn (Python version 3.11) implementations unless otherwise specified (Table 2); systematic hyperparameter tuning via grid search was performed only for the SVM classifier (kernel, C, gamma, and tolerance selected via 5-fold cross-validation on the respective training data), while hyperparameters for all other feature selection and classification methods were fixed at default or manually specified values without further tuning. Because the best-performing feature selection–classifier combination was selected based on test-fold performance itself rather than through a further nested validation step, the reported performance metrics likely represent an optimistic estimate of generalization performance.

Confidence intervals of 95% for accuracy, F1-score, and AUC were calculated across the three independent cross-validation runs using a t-distribution-based approach: each interval was defined as the mean of the three runs ± the margin of error, where the margin of error was calculated as the two-sided critical t-value (α = 0.05, 2 degrees of freedom) multiplied by the standard error (standard deviation divided by the square root of the number of runs, *n* = 3). For metrics that were numerically identical across all three runs, the standard deviation was zero or near-zero, likely reflecting rounding within the underlying analysis pipeline, resulting in a correspondingly narrow or zero-width confidence interval; these results are reported alongside the corresponding mean performance metrics.

## 3. Results

### 3.1. Demographics

Applying the CVS definition adopted for this study, angiographically confirmed CVS was documented in 57 patients, while DCI developed in 45 patients after aneurysm rupture (Table 3).

For the assessment of CSF infection, microbiological pathogen detection in the CSF obtained via EVD was considered. In total, 134 EVD were documented; 23 had been inserted externally prior to transfer (mean duration 9.5 days), whereas 115 drains were inserted or exchanged at our institution (mean duration 9.4 days). Microbiological evidence of a pathogen in the CSF was identified in 28 patients. During their ICU stay, 116 patients received antibiotic therapy. Piperacillin/tazobactam was the most frequently administered agent (101 of 120 antibiotic courses with a documented regimen, 84%), which is consistent with its role as first-line empirical therapy for suspected pneumonia or urinary tract infection. Meropenem (32 patients, 27%) and vancomycin (17 patients, 14%) were the next most common agents, most frequently administered in combination, reflecting escalation to the institutional empirical regimen for suspected CNS infection alongside continued treatment of a concurrent extracranial infection. Additional agents (e.g., ceftriaxone, ampicillin, flucloxacillin, fosfomycin, and linezolid) were used in smaller numbers of patients, generally reflecting antibiogram-guided adjustment once a causative pathogen had been identified.

**Figure 1:**
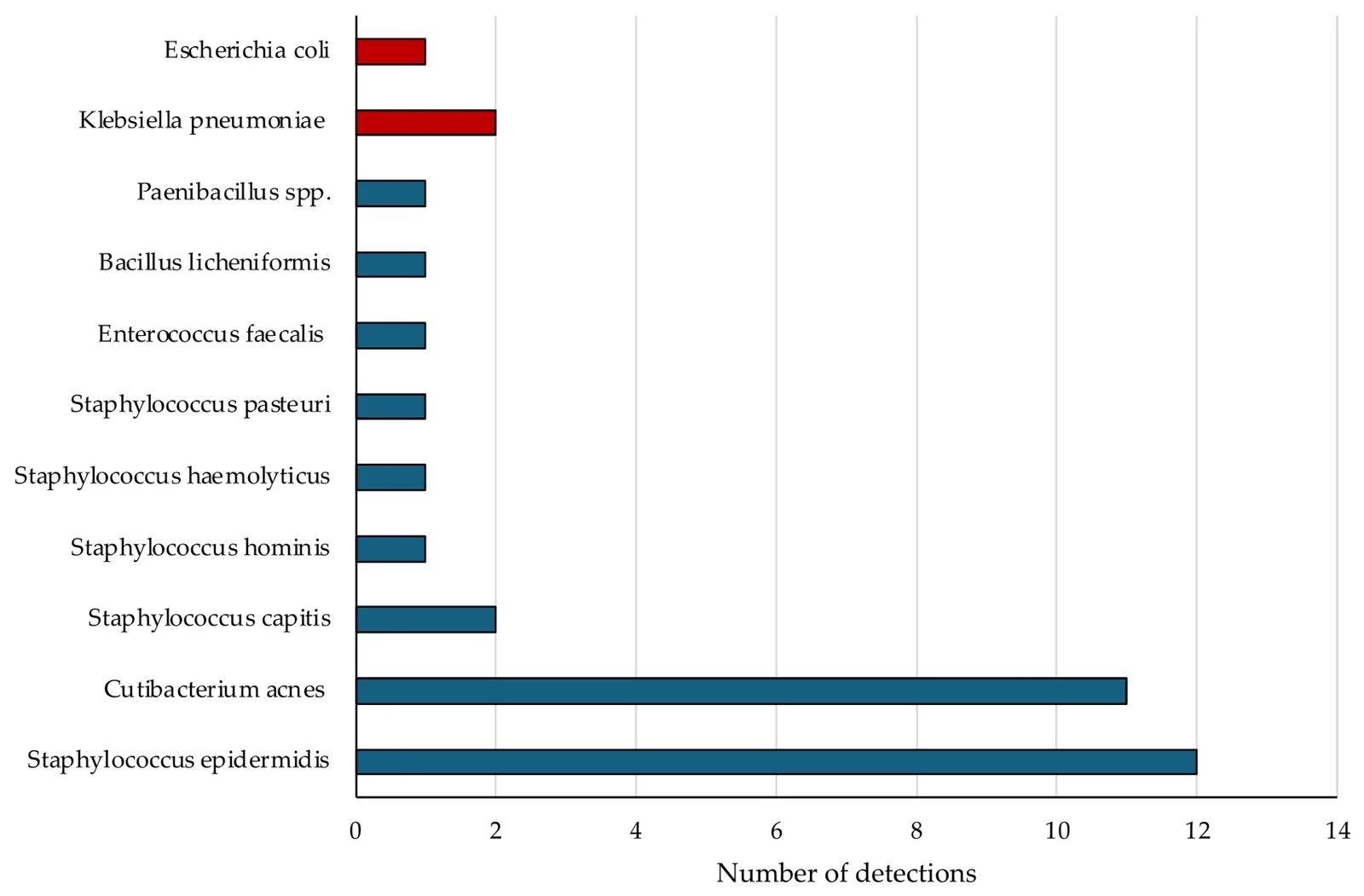
Distribution of bacterial species detected in CSF cultures. Horizontal bar chart showing the frequency of microorganisms identified in CSF samples obtained from external ventricular or lumbar drains in patients with aneurysmal subarachnoid hemorrhage during intensive care. Blue bars indicate Gram-positive bacteria; red bars indicate Gram-negative bacteria. Polymicrobial cultures contributed to the count of each detected species. Most isolates represented Gram-positive skin commensals, predominantly *Staphylococcus epidermidis* and *Cutibacterium acnes*, whereas *Gram-negative organisms* such as *Klebsiella pneumoniae* and *Escherichia coli* were uncommon.

Overall, 45 pathogen detections were recorded. One patient experienced two distinct infections and another patient three. Most isolates represented Gram-positive skin commensals, predominantly *Staphylococcus epidermidis* and *Cutibacterium acnes*, whereas *Gram-negative organisms* were uncommon. The complete distribution of detected microorganisms is shown in Figure 1.

### 3.2. Univariate Comparison of Predictors by Outcome Group

To complement the machine learning results, the clinical, laboratory, and cerebrospinal fluid parameters identified as the most relevant predictors were additionally compared between outcome groups using conventional univariate statistics. Continuous variables were tested with Welch’s *t*-test or the Mann–Whitney U test, depending on distribution normality assessed by the Shapiro–Wilk test, and are presented as mean ± standard deviation or median [interquartile range], respectively. Categorical variables were compared using the chi-square test or Fisher’s exact test where expected cell counts were below five. For DCI, patients who developed DCI differed significantly from those who did not with respect to antibiotic therapy and history of thrombosis, while several laboratory trends (albumin, RDW, and maximum CSF nucleated cell count) approached but did not reach significance (Table 4).

For functional outcome, patients with an unfavorable outcome (mRS 3–6) showed significantly higher mean AST and LDH levels and a significantly lower proportion of CSF mononuclear cells than patients with a favorable outcome (mRS 0–2), whereas the remaining candidate predictors did not reach statistical significance in univariate testing (Table 5). These findings indicate that not all predictors selected by the machine learning models are individually significant on univariate testing, which is consistent with the models capturing multivariate and non-linear relationships among predictors that are not fully reflected in pairwise group comparisons.

### 3.3. Machine Learning Approach

#### 3.3.1. Occurrence of DCI

**Figure 2:**
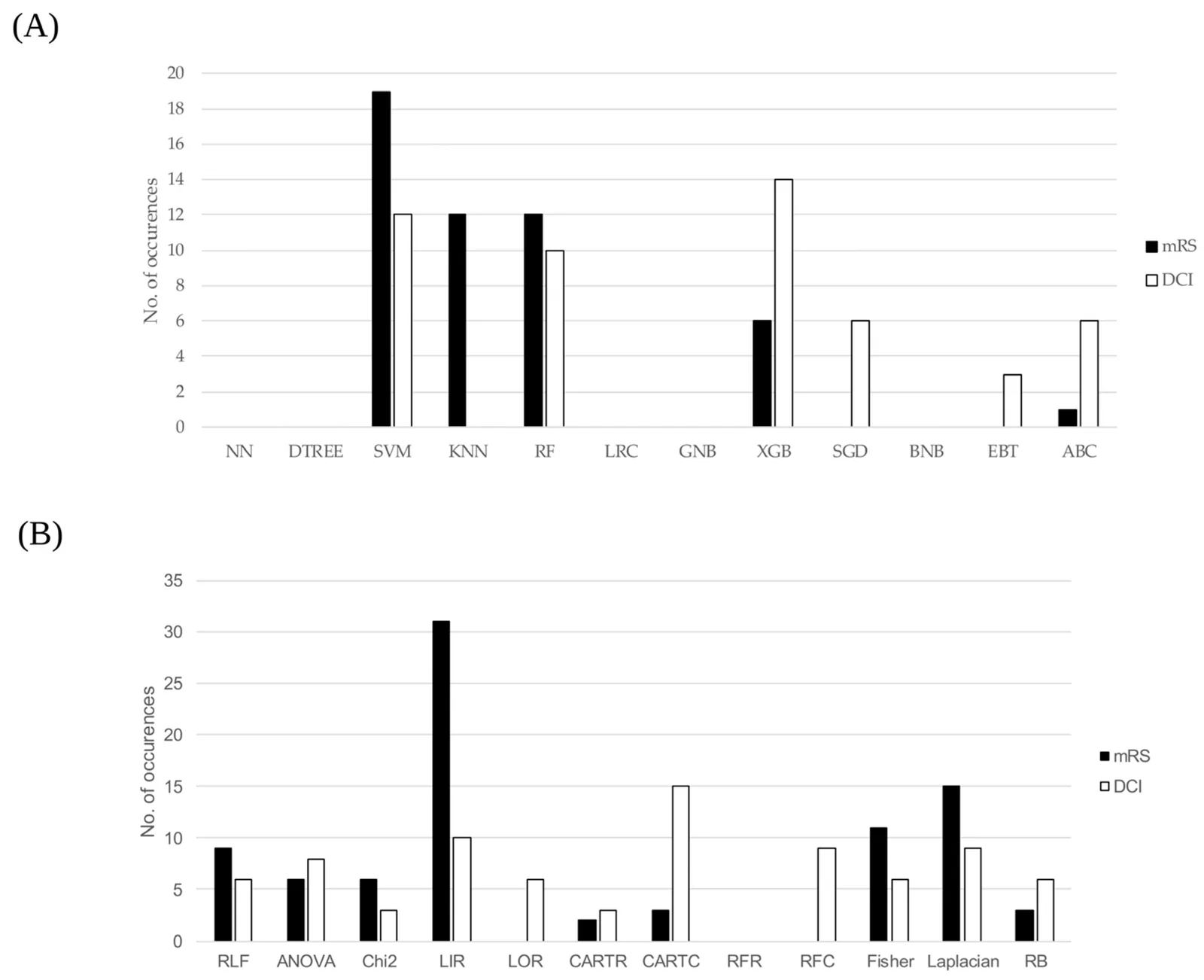
Frequency of best-performing classifiers and feature selection methods in the machine learning analysis. In both panels, dark bars represent the mRS (functional outcome) prediction task and light bars represent the DCI prediction task. (**A**) Frequency with which individual classification algorithms produced the best-performing models for predicting DCI and functional outcome at discharge (mRS). (**B**) Frequency with which different feature selection methods identified the highest-ranking feature subsets; for each approach, multiple classifiers were trained and evaluated, and the number of selected predictors was limited to ten to reduce dimensionality and inter-feature correlation.

For the prediction of DCI, classification algorithms such as XGB, SVM, and RF most consistently achieved superior performance. Among the feature selection techniques, CARTC, LIR, ANOVA, RFC, and the Laplacian Score most frequently yielded the highest-ranking feature subsets (Figure 2).

DCI was modeled as a binary outcome. Across three independent runs, the models demonstrated stable and reproducible performance, achieving an accuracy of 96% in each run. The corresponding F1-score was 0.96 in all runs. The area under the receiver operating characteristic curve ranged from 0.9735 to 0.9839, with a mean ROC AUC of 0.9797 (95% CI: 0.966–0.993), indicating excellent discriminative ability (Table 6).

The most relevant predictors comprised both categorical and laboratory-derived variables. Among the categorical features were the administration of antibiotic therapy, the type of aneurysm repair, a history of thrombosis, and the need for EVD revision.

Laboratory parameters obtained from peripheral blood included the mean albumin concentration, mean RDW, mean INR, and minimum aspartate aminotransferase levels. In addition, CSF parameters, namely the mean CSF glucose, the mean CSF lactate, the maximum count of nucleated cells, and the presence of cellular debris on microscopy, also emerged as relevant predictors.

The consistently high performance across all runs suggests robust generalizability and minimal overfitting within the applied cross-validation framework. The near-perfect ROC AUC values indicate excellent discrimination between patients who developed DCI and those who did not.

To characterize clinical operating performance, sensitivity, specificity, positive predictive value (PPV), and negative predictive value (NPV) were derived from the confusion matrix of the best-performing model in each run, evaluated on the class-balanced test folds used during cross-validation. Because these folds were balanced through oversampling and undersampling rather than reflecting the true prevalence of DCI in the underlying cohort (23.6%), the reported PPV and NPV should be interpreted as operating characteristics under balanced class conditions rather than as directly applicable real-world predictive values; PPV in particular would be expected to decrease at the lower prevalence encountered in unselected clinical populations. Sensitivity was 0.96 in the first run and 0.95 in the second and third runs, while specificity remained constant at 0.97 across all three runs. PPV was consistently 0.97, and NPV corresponded to 0.96 in the first run and 0.95 in the subsequent runs. Averaged across all three runs, the model achieved a mean sensitivity of 0.95, specificity of 0.97, PPV of 0.97, and NPV of 0.95.

#### 3.3.2. Functional Outcome

To predict functional outcome at discharge as defined by the mRS, a supervised machine learning framework was applied. Among the evaluated classifiers, SVM, KNN, and RF consistently demonstrated the strongest predictive performance. Regarding feature selection, LIR, the Laplacian Score, the Fisher scale, and RLF most frequently identified the top-ranked feature sets (Figure 2).

Model performance was highly stable across three independent runs. In each run, an accuracy of 94.8% was achieved, accompanied by a consistent F1-score of 0.95. The area under the receiver operating characteristic curve varied between 0.9694 and 0.9762, with a mean ROC AUC of 0.9723 (95% CI: 0.964–0.981), reflecting excellent discriminatory capacity (Table 7).

The most relevant predictors of functional outcome, as assessed by the mRS, comprised both clinical–categorical and laboratory-derived variables. Among the categorical features were the occurrence of CVS, the number of spasmolysis procedures performed, pre-admission anticoagulant therapy, microbiological pathogen detection in the CSF, and pathogen type.

Peripheral blood parameters contributing to outcome prediction included minimum aPTT, mean fibrinogen levels, mean aspartate aminotransferase levels, and mean lactate dehydrogenase levels. Relevant CSF variables comprised the minimum proportion of mononuclear cells, the minimum leukocyte count, and the maximum glucose concentration.

The stable performance across all validation runs indicates good generalizability of the model and suggests minimal overfitting within the applied cross-validation framework. The consistently high ROC AUC values demonstrate excellent discrimination between patients with favorable and unfavorable functional outcome at discharge.

Sensitivity, specificity, PPV, and NPV for the best-performing functional outcome model were derived from the class-balanced test folds; as above, these values should be interpreted in the context of the balanced evaluation setting rather than the true prevalence of unfavorable outcome in the cohort. The model achieved a sensitivity of 0.98, specificity of 0.92, PPV of 0.92, and NPV of 0.98 consistently across all three cross-validation runs and on average.

## 4. Discussion

This study applied a comprehensive machine learning framework to investigate the role of CNS infection-related parameters in the development of DCI and functional outcomes in patients with aSAH. The models achieved excellent predictive performance, with a mean AUC of 0.9797 for DCI prediction and 0.9723 for functional outcome at discharge. Notably, microbiological pathogen detection in the CSF emerged as a top-ranked predictor for functional outcome, alongside established clinical variables such as angiographically confirmed CVS and laboratory parameters.

Prior prognostic models for DCI and outcome after aSAH have relied predominantly on admission variables, imaging findings, and CVS status, with CNS infection typically treated as an incidental complication rather than a systematically modeled predictor. To the best of our knowledge, this is among the first machine learning studies to integrate longitudinal CSF microbiological and inflammatory parameters alongside systemic laboratory and clinical variables within a unified predictive framework for both DCI and functional outcome. This addresses an important gap, as infection-related processes are increasingly implicated in secondary brain injury after aSAH yet have rarely been incorporated as first-class predictors in existing outcome models. Our findings extend the existing evidence base by demonstrating that CNS infection-related parameters carry predictive value comparable to, and in the case of functional outcome exceeding, established CVS-related variables.

### 4.1. CNS Infection and Microbiology

In our cohort, 134 EVD were documented, with microbiological evidence of a pathogen in the CSF identified in 28 patients (20.9% of EVD patients). This pathogen detection rate falls within the upper range of reported EVD-associated infection rates of 0–22% [32,33,34], although, as detailed in Section 2.3, this definition does not distinguish true infection from contamination. The predominance of coagulase-negative staphylococci and *Cutibacterium acnes* in our cohort is consistent with the literature, which identifies skin flora as the most common causative organisms in EVD-associated ventriculitis [32,33,34,35]. *Staphylococcus* spp. and other Gram-positive organisms have been reported as the primary pathogens in most series, although Gram-negative bacteria (including *Klebsiella pneumoniae* and *Escherichia coli*, both identified in our cohort) are increasingly recognized and associated with higher mortality [35,36]. The identification of microbiological pathogen detection as a key predictor of both DCI and functional outcome is consistent with an association between CNS infection-related processes and secondary brain injury, potentially involving neuroinflammatory, microvascular thrombosis, and impaired cerebral autoregulation [6,37,38]. However, because our pathogen detection variable cannot identify contamination, this association should be regarded as hypothesis-generating rather than as confirmatory evidence of a causal role for true CNS infection.

### 4.2. Predictors of DCI

The most relevant DCI predictors spanned categorical, clinical, and laboratory domains: antibiotic therapy, aneurysm treatment modality, history of thrombosis, and EVD revision; peripheral parameters including mean albumin, RDW, INR, and minimum aspartate aminotransferase; and CSF variables including glucose, lactate, nucleated cell count, and cellular debris.

Notably, CVS-related variables were absent from the DCI model. Although traditionally considered central to DCI pathogenesis, this absence is consistent with the evolving understanding that DCI reflects microvascular dysfunction, inflammation, and metabolic disturbance rather than large-vessel CVS alone: CVS occurs in up to 70% of aSAH patients, whereas DCI develops in only 20–30% [3,4], and CVS reduction does not consistently improve outcomes [5,39]. Contemporary frameworks, including the 2023 AHA/ASA guidelines, emphasize multifactorial mechanisms such as microthrombosis, cortical spreading depolarizations, and impaired autoregulation [39,40,41], suggesting that the neurocritical care course may provide more relevant risk-stratification information than CVS status alone.

Several peripheral blood parameters reflect distinct but converging pathophysiological axes. Mean albumin, as demonstrated by Burzyńska et al. [42], likely reflects systemic inflammation, capillary leak, and illness severity. Mean RDW, previously linked to DCI by Wiśniewski et al. (AUC 0.924 in combination with CSF isoprostane) [43] and Ignacio et al. [44], reflects oxidative stress and erythrocyte fragmentation. Mean INR aligns with the established role of coagulopathy and microthrombosis in DCI, including recent nomogram-based evidence implicating aPTT as an independent predictor [5,39,45]. Minimum aspartate aminotransferase plausibly reflects hepatic stress, hemolysis, or multiorgan dysfunction; although understudied as a standalone DCI marker, its biological plausibility is supported by its association with systemic inflammatory responses in critical illness.

CSF-derived variables constituted a clinically distinct predictor category. CSF glucose and lactate have been validated as DCI biomarkers by Messina et al., who similarly found no significant CVS–DCI correlation after multivariate adjustment [46]; lactate elevation likely reflects anaerobic metabolism from early brain injury, while glucose alterations may indicate blood–brain barrier dysfunction or increased cerebral metabolic demand [3,47,48]. Maximum nucleated cell count and cellular debris on microscopy likely capture the intensity of the neuroinflammatory response and blood breakdown product burden, which is consistent with the temporal correlation between CSF/systemic inflammation and DCI [3], and may additionally serve as proxies for infectious complications such as ventriculitis—directly linking to the antibiotic therapy predictor described below.

The emergence of antibiotic therapy and EVD revision as predictors likely reflects infectious complications (ventriculitis, pneumonia, and sepsis) and a more complicated neurocritical care course (drainage failure, catheter obstruction, and secondary hemorrhage), which is consistent with intracranial infection as an independent DCI risk factor in meta-analytic data [7]. Aneurysm treatment modality has been variably associated with DCI risk; a recent meta-analysis found no independent association, suggesting that its emergence here may reflect confounding by indication (e.g., more complex aneurysms or higher-grade hemorrhage preferentially treated surgically) or treatment-specific inflammatory responses [49]. History of thrombosis is a novel but biologically plausible predictor: a pre-existing prothrombotic state could lower the threshold for microvascular occlusion in the post-SAH inflammatory milieu [5,39], warranting further investigation in larger cohorts.

### 4.3. Predictors of Functional Outcome

The most relevant predictors of functional outcome comprised CVS occurrence, number of spasmolysis procedures, pre-admission anticoagulant therapy, CSF pathogen detection and type, peripheral parameters (minimum aPTT, mean fibrinogen, mean aspartate aminotransferase, and mean lactate dehydrogenase), and CSF parameters (minimum mononuclear cell proportion, minimum leukocyte count, and maximum glucose).

In contrast to the DCI model, CVS and spasmolysis procedure count featured prominently, which is consistent with the established role of angiographic CVS in morbidity and mortality after aSAH [2,40] and the 2023 AHA/ASA recommendation of endovascular spasmolysis for medically refractory CVS [40]. The number of spasmolysis procedures likely surrogates for CVS severity, treatment refractoriness, and cumulative ischemic burden; its prominence here—despite the absence of CVS from the DCI model—suggests that the downstream consequences of CVS affect neurological recovery even where the CVS–DCI relationship is not straightforward [2].

Pre-admission anticoagulant therapy warrants cautious interpretation given mixed evidence: large cohort and case–control studies have found no independent association between anticoagulant use and outcome after multivariable adjustment [50,51,52]. Its emergence here may reflect confounding by the comorbidities necessitating anticoagulation, iatrogenic coagulopathy affecting early hemorrhage management, or interaction with aSAH-associated coagulation perturbations not fully captured by conventional adjustment.

CSF pathogen detection and type underscore the role of infectious complications in shaping recovery: nearly half of aSAH patients develop nosocomial infections, with specific organisms (*Staphylococcus*, *Klebsiella*, and *Enterococcus*) linked to poor long-term outcome [9], which is consistent with the 2023 AHA/ASA emphasis on aseptic technique and antibiotic-impregnated catheters [40]. That pathogen type independently contributes to outcome prediction suggests that organism-specific virulence and inflammatory profiles differentially affect neuroinflammation and recovery; notably, Togni et al. found no significant difference in CSF cell count dynamics between microbiologically confirmed ventriculostomy-related infection and uninfected patients, underscoring the added prognostic value of microbiological culture data beyond routine CSF parameters [24]. CSF leukocyte counts follow a non-linear course over the first 14 days with a peak at days four to eight, and more rapid erythrocyte clearance is associated with favorable outcome [24], while elevated CSF white and red blood cells are linked to DCI [53]; a lower minimum mononuclear cell proportion may indicate a persistently granulocyte-predominant and less reparative inflammatory profile. Maximum CSF glucose is consistent with known associations between CSF glucose alterations and outcome [54], potentially reflecting systemic hyperglycemia—itself a predictor of poor outcome—or blood–brain barrier glucose transport dysfunction.

Peripheral blood parameters again reflected converging pathophysiological axes. Minimum aPTT aligns with evidence linking unfavorable outcome to a hypercoagulable state on thromboelastometry [55], suggesting microthrombosis-driven secondary injury. Mean fibrinogen is consistent with its established prognostic role via the fibrinogen-neutrophil/lymphocyte ratio score [56,57] and albumin-to-fibrinogen ratio [58], with a possible U-shaped relationship in which both low and high levels are deleterious [59]. Mean aspartate aminotransferase plausibly reflects hepatic stress or multiorgan dysfunction, and mean lactate dehydrogenase is supported by evidence linking peak ICU values (threshold > 272 IU/L) to unfavorable three-month outcome [60] and to mortality in critically ill stroke patients [61]. Although the DCI and functional outcome models draw on overlapping physiological domains—infection and inflammation, coagulation, and CSF composition—the specific predictors identified for each outcome differ substantially and overlap only partially (e.g., aspartate aminotransferase and CSF glucose, captured via different aggregation statistics for each outcome). DCI predictors were dominated by markers of infection, coagulopathy, and CSF inflammatory burden, whereas functional outcome predictors additionally incorporated CVS-related and coagulation-intensity variables not selected for DCI. This pattern indicates that, while DCI and functional outcome share related pathophysiological underpinnings, they are not biologically identical endpoints and should not be assumed to share a common predictor profile; the two outcomes are therefore discussed as related but distinct processes throughout this section. The analysis deliberately relied on routinely available parameters already captured during standard neurocritical care rather than composite inflammatory indices (e.g., neutrophil-to-lymphocyte ratio) or specialized biomarkers requiring additional testing (e.g., HbA1c, prealbumin). Differential leukocyte counts were included as individual variables, allowing the algorithms to capture ratio-like effects without a prespecified index; established ratios such as the neutrophil-to-lymphocyte and fibrinogen-neutrophil-to-lymphocyte ratio scores have independently been validated as DCI/outcome predictors elsewhere [58,59], supporting the plausibility of our underlying components. Similarly, glucose and albumin were chosen because they are part of the standard biochemical panel, whereas HbA1c (needed for the stress hyperglycemia ratio) is not routinely drawn at admission and prealbumin is not part of our panel; both would introduce substantial missingness if applied retrospectively. We acknowledge these composite and specialized markers are biologically plausible and recommend their prospective inclusion in future studies.

### 4.4. CNS Infection, Inflammation, and Thromboinflammation as a Unifying Pathway

Considered together, the predictor profiles identified for DCI and functional outcome converge on a shared thromboinflammatory axis, with CNS infection-related processes, systemic and intrathecal inflammation, and coagulation disturbance all represented among the identified predictors. CNS infection-related processes—reflected by pathogen detection, antibiotic therapy, and CSF inflammatory parameters, without formal discrimination between true infection and contamination—were associated with markers of neuroinflammation (neutrophil and monocyte activation, blood–brain barrier permeability, and cytokine release) that have been biologically linked to coagulation activation in the prior literature [6,37,38]. The altered INR, aPTT, and fibrinogen values observed among our predictors are similarly consistent with a prothrombotic state that has been associated with microvascular thrombosis and DCI independent of large-vessel CVS in prior work [5,39]. Cerebral autoregulatory and microcirculatory impairment represent a biologically plausible pathway potentially linking these infection- and coagulation-related parameters to DCI and long-term functional outcome, although this cannot be confirmed from the present data. Given the observational, retrospective design of this study—in which predictors and outcomes were not measured at strictly defined, temporally ordered time points, and residual confounding by unmeasured factors (e.g., illness severity, treatment intensity) cannot be excluded—these findings should be interpreted as associations rather than as evidence of causal or mechanistic relationships. We therefore present this thromboinflammatory framework as a hypothesis for future prospective, mechanistically oriented studies rather than as an established causal pathway; such studies would be needed before considering anti-inflammatory or infection-prevention strategies as adjuncts to conventional CVS-directed therapy.

### 4.5. Comparison with Prior Machine Learning Studies

Our models achieved a mean AUC of 0.98 for DCI and 0.97 for functional outcome, exceeding most previously reported machine learning approaches for aSAH. For DCI prediction, a recent meta-analysis of 48 studies comprising 71 models reported a pooled C-index of 0.79 in training and 0.77 in validation sets [62]. Individual studies have re-ported lower AUCs using clinical data alone, CT-derived features, or a combination thereof: Savarraj et al. reported an AUC of 0.75 using clinical data collected within three days of admission [19]; Hu et al. achieved an AUC of 0.86 with Random Forest and artificial Neural Network models incorporating CT attenuation and blood cell counts [18]; Yin et al. reported AUCs of 0.81–0.95 using CT perfusion parameters, with CatBoost performing best [20]; and Ge et al. reported an AUC of 0.79 using Random Forest with admission mRS, D-dimer, and neutrophil-to-lymphocyte ratio as key features [22]. For functional outcome, reported AUCs range from 0.85 to 0.96 across comparable approaches: 0.85 (discharge) and 0.89 (three-month) with clinical data [19]; 0.86–0.96 with CT perfusion-based CatBoost models [20]; 0.85 with a feedforward artificial Neural Network for six-month outcome [63]; 0.85 with a “Late” prediction model incorporating secondary complications [64]; 0.90 with a deep learning model based on pre-treatment data [65]; and 0.87 (training) and 0.85 (validation) with XGBoost for 180-day outcome [66]. The consistently high performance observed in our models for both endpoints, together with stability across three independent training runs, suggests robust performance and limited overfitting within the applied cross-validation framework; nonetheless, the higher AUCs relative to prior work should be interpreted in light of the single-center design.

### 4.6. Clinical Implications

These findings carry several concrete implications for neurocritical care practice. First, the prominence of infection-related variables (antibiotic therapy, CSF inflammatory markers, and pathogen detection and type) underscores the importance of strict infection-prevention protocols, including antiseptic EVD insertion and maintenance bundles, antibiotic-impregnated catheters, and vigilant surveillance for ventriculostomy-related infection, as a potential lever for reducing both DCI risk and unfavorable functional outcome. Second, because the identified predictors rely on laboratory and CSF parameters that are already routinely collected during ICU care, they could in principle be embedded into an automated risk-stratification tool integrated with the electronic health record to support ongoing risk assessment during the neurocritical care course. Given that many predictors are measured concurrently with or after DCI onset (see Limitations), such a tool would characterize the overall clinical trajectory rather than provide early warning ahead of DCI onset. Third, the thromboinflammatory predictor profile identified here supports evaluating anti-inflammatory or infection-prevention strategies as adjuncts to conventional CVS-directed DCI prophylaxis, a hypothesis that would need to be tested in prospective interventional studies. Finally, the high and stable predictive performance observed across cross-validation runs illustrates the potential of data-driven, multivariable models to support individualized risk assessment in neurocritical care, although prospective, multicenter validation is required before such tools could be considered for clinical deployment.

### 4.7. Limitations

This study has several limitations. First, its retrospective single-center design may limit generalizability, and external validation in multicenter cohorts is required. Second, although the cohort size is comparable to similar studies, the number of events remains limited (45 DCI events and 28 CSF pathogen detections among 191 patients), constraining the statistical power of subgroup analyses and increasing the risk that cross-validated performance estimates are optimistic. Together with the retrospective, single-center design and the absence of external validation, these constraints mean that the present findings should be regarded as hypothesis-generating rather than as evidence supporting immediate clinical deployment, and require confirmation in prospective, adequately powered, multicenter cohorts. Third, CNS infection was not defined using a standardized composite clinical definition but rather assessed through individual parameters, which may have introduced misclassification. Fourth, the retrospective design limits conclusions regarding temporal and causal relationships between predictors and DCI. Fifth, and most importantly, because clinical, laboratory, and CSF variables were aggregated as mean, minimum, or maximum values across the entire ICU stay rather than restricted to baseline or to a defined pre-DCI window, several identified predictors—including antibiotic therapy, EVD revision, number of spasmolysis procedures, and serial CSF or laboratory values—may reflect events or measurements occurring concurrently with or after DCI onset rather than strictly preceding it. The present dataset does not include values time-stamped relative to individual DCI onset, and this temporal ambiguity could not be resolved within the current retrospective design. Consequently, the reported performance should be interpreted as reflecting associations across the neurocritical care course rather than early, baseline-only prediction of DCI. Future prospective studies with time-stamped data and landmark or dynamic modeling approaches restricted to pre-DCI information are needed to establish genuine early predictive performance. Sixth, the use of aggregated longitudinal variables may additionally reduce temporal resolution and interpretability, and despite cross-validation, some degree of overfitting cannot be excluded. Seventh, hyperparameters for most feature selection and classification algorithms were set to default or manually specified values rather than systematically tuned (Table 2), with the exception of the SVM classifier, for which hyperparameters were selected via grid search; and the best-performing feature selection–classifier combination in each fold was selected based on that fold’s own test performance rather than through an additional nested validation layer; both factors likely contribute further to optimistic performance estimates and should be addressed in future work through nested cross-validation for model selection and systematic hyperparameter optimization. Class imbalance (23.6% DCI events) was addressed through a combination of oversampling and undersampling applied to the training data prior to model fitting, in addition to stratified fold splitting; future analyses could explore and compare alternative balancing strategies (e.g., SMOTE, class-weighting) to assess their impact on model performance and calibration. Finally, the absence of CVS-related variables among top predictors may reflect model-specific feature selection rather than biological irrelevance. This approach cannot reliably distinguish true CNS infection (ventriculitis/meningitis) from culture contamination, particularly given the predominance of cutaneous commensal organisms in our cohort. Consequently, statements regarding the “role of CNS infection” in this study should be interpreted as pertaining to CSF pathogen detection and related surrogate markers rather than to confirmed clinical infection. Eighth, we did not compare the machine learning models against a simpler baseline approach, such as conventional Logistic Regression using the same predictors, nor did we perform internal validation on a separate hold-out set that was entirely excluded from feature selection and model training; both would help clarify the added value of the applied machine learning framework relative to conventional statistical modeling and provide a further safeguard against optimistic performance estimates. We recommend that future studies incorporate a Logistic Regression benchmark and a strictly independent hold-out set, alongside prospective multicenter validation, to comprehensively establish the incremental value and generalizability of the proposed models.

## 5. Conclusions

DCI and functional outcome after aSAH are best understood as multifactorial processes shaped by the interplay of systemic inflammation, coagulation disturbances, infectious complications, and intracranial metabolic stress. Using a comprehensive machine learning approach, this study identifies a set of clinical, laboratory, and CSF-derived predictors that extend beyond traditional CVS-centered paradigms.

These findings highlight the importance of the neurocritical care course in determining both DCI risk and functional outcome, supporting the integration of systemic and intrathecal biomarkers into risk stratification. Given the retrospective, single-center design, modest sample size, and absence of external validation, these findings should be regarded as hypothesis-generating rather than as evidence ready for clinical deployment. Machine learning-based models may eventually provide a valuable tool for individualized prediction and management in neurocritical care, but prospective, adequately powered multicenter studies with external validation are required to confirm these findings before they can inform clinical practice or the evaluation of targeted therapeutic strategies.

## Figures and Tables

**Table 1:** Patient-related and aneurysm-related epidemiological characteristics of the cohort.

**Patient-Related Epidemiological Characteristics**		
	**N**	**%**
Sex		
Male	58	30.4
Female	133	69.6
Age		
<70 years	151	79.1
≥70 years	40	20.9
Mean	56.5	
Vascular risk factors		
Hypertension	117	61.3
Diabetes Type 2	13	6.8
Hyperlipidemia	38	19.9
Nicotine abuse	76	39.8
Alcohol abuse	21	11
Obesity	29	15.2
Stroke	17	9
Peripheral arterial diseases	4	2.1
Coronary heart disease	5	2.6
Myocardial infarction	2	1.1
Hunt and Hess scale		
Grade 1	7	3.7
Grade 2	83	43.5
Grade 3	38	19.9
Grade 4	15	7.9
Grade 5	46	24.1
Missing information	2	
**Aneurysm-Related Epidemiological Characteristics**		
	**N**	**%**
Number of aneurysms		
Single	137	71.7
Multiple	54	28.3
Localization		
ACA	2	1.1
ACOM	65	34
Pericallosal artery	5	2.6
MCA	37	19.4
ICA	31	16.2
PCOM	13	6.8
Basilar artery	17	8.9
PICA	11	5.8
Others	10	5.2
Aneurysm treatment		
Endovascular treatment	113	59.2
Surgical treatment	59	30.9
No treatment	19	9.9

**Table 2:** Parameter settings used for the twelve feature selection methods and twelve classification algorithms. Unmarked methods used default library implementations.

Abbreviation	Method	Parameters
Feature selection methods		
RLF	ReliefF	n_neighbors = 20
ANOVA	Analysis of Variance	score_func = f_classif, k = ‘all’
Chi2	Chi-squared Test	score_func = chi2, k = ‘all’
LIR	Linear Regression	default settings
LOR	Logistic Regression	max_iter = 1000
CARTR	CART Regression	default settings
CARTC	CART Classification	default settings
RFR	Random Forest Regression	random_state = 42
RFC	Random Forest Classification	random_state = 42
Fisher	Fisher Scale	default settings
Laplacian	Laplacian Score	metric = euclidean, neighbor_mode = knn, weight_mode = heat kernel, k = 2, t = 1
RB	Gradient Boosting	default settings
Classification methods		
NN	Neural Network	solver = lbfgs, alpha = 1 × 10^−5^, hidden_layer_sizes = (150, 10), random_state = 1
DTREE	Decision Tree (medium)	max_depth = 5, min_samples_split = 10, min_samples_leaf = 5
SVM	Support Vector Machine	grid search (5-fold CV) over C = [0.1, 0.5, 0.8, 1, 10, 100], gamma = [0.0001, 0.001, 0.01, 1], tol = [0.001, 0.01, 0.1], kernel = [rbf, poly, linear, sigmoid]
KNNs	k-Nearest Neighbors	n_neighbors = 5
RF	Random Forest	n_estimators = 1000, max_depth = 10, random_state = 0
LRC	Logistic Regression	default settings
GNB	Gaussian Naive Bayes	default settings
XGB	eXtreme Gradient Boosting	n_estimators = 100, max_depth = 6, learning_rate = 0.1, eval_metric = logloss, random_state = 0
SGD	Stochastic Gradient Descent	loss = log loss, random_state = 42, cv = 5
BNB	Bernoulli Naive Bayes	threshold = 0.0
EBTs	Ensemble Bagged Trees	base estimator = Decision Tree, n_estimators = 10, random_state = 0
ABC	AdaBoost	base estimator = Decision Tree (max_depth = 1), n_estimators = 100, random_state = 0

**Table 3:** Frequency of angiographically confirmed CVS and DCI in the aSAH cohort.

Parameter	Yes (N/%)	No (N/%)
Angiographically confirmed CVS	57/29.9	134/70.1
DCI	45/23.6	146/76.4

**Table 4:** Univariate comparison of key clinical, laboratory, and cerebrospinal fluid parameters between patients with and without DCI.

Variable	DCI	No DCI	*p*-Value
Antibiotic therapy, n (%) *	40	78	0.0001
History of thrombosis, n (%) **	6	6	0.039
Aneurysm treatment * (clipping/coiling/conservative/dome protection)	12/31/1/1	48/82/12/1	0.272
EVD revision, n (%) *	32	82	0.061
Mean albumin (g/L) ***	32.7	33.9	0.074
Mean RDW (%) ****	13.9	13.6	0.058
Mean INR ****	1.10	1.09	0.277
Minimum AST (µmol/s·L) ****	0.27	0.28	0.527
Mean CSF glucose (mmol/L) ***	3.96	4.03	0.754
Mean CSF lactate (mmol/L) ****	3.73	3.35	0.845
Maximum CSF nucleated cell count ****	1128	455	0.065
Cellular debris, n ** (detected vs. occasional)	1	6	0.666

* χ^2^, ** Fisher’s exact, *** Welch t, **** Mann–Whitney U.

**Table 5:** Univariate comparison of key clinical, laboratory, and cerebrospinal fluid parameters between patients with favorable and unfavorable functional outcomes.

Variable	mRS 3–6	mRS 0–2	*p*-Value
Angiographically confirmed CVS, n (%) *	42	15	0.506
Number of spasmolysis procedures ****	0	0	0.202
Pre-admission anticoagulation, n (%) **	4	0	0.301
CSF pathogen detection, n (%) *	1	9	0.575
Minimum aPTT (sec) ****	24.9	24.6	0.442
Mean fibrinogen (g/L) ****	3.31	3.28	0.552
Mean AST (µmol/s·L) ****	0.69	0.49	<0.0001
Mean LDH (µmol/s·L) ****	3.60	3.10	<0.0001
Minimum CSF mononuclear cells (%) ****	25.9	36.9	0.0006
Minimum CSF leukocyte count ****	23	40	0.418
Maximum CSF glucose (mmol/L) ****	4.60	4.29	0.202

* χ^2^, ** Fisher’s exact, **** Mann–Whitney U.

**Table 6:** Classification performance for DCI prediction. Accuracy, F1-score, and ROC AUC are presented per run and averaged across three independent runs including the confidence interval.

Run	Accuracy	F1-Score	ROC AUC
1. Run	0.96	0.96	0.9735
2. Run	0.96	0.96	0.9817
3. Run	0.96	0.96	0.9839
Mean	0.96	0.96	0.9797
CI	0.951–0.967	0.96–0.96	0.966–0.993

**Table 7:** Performance metrics of the best-performing machine learning classifiers for predicting functional outcome at discharge. Accuracy, F1-score, and area under the ROC curve are reported for three independent runs and as mean values including the confidence interval. Favorable outcome was defined as mRS 0–2, and unfavorable outcome as mRS 3–6.

Run	Accuracy	F1-Score	ROC AUC
1. Run	0.9478	0.95	0.9762
2. Run	0.9478	0.95	0.9714
3. Run	0.9478	0.95	0.9694
Mean	0.9478	0.95	0.9723
CI	0.948–0.948	0.95–0.95	0.964–0.981

## Data Availability

The dataset supporting the conclusions of this article is included within the article.

## Abbreviations

The following abbreviations are used in this manuscript:

aSAH	Aneurysmal subarachnoid hemorrhage
AUC	Area under the curve
CNS	Central nervous system
CSF	Cerebrospinal fluid
CT	Computed tomography
CVS	Cerebral vasospasm
DCI	Delayed cerebral ischemia
ICU	Intensive care unit
GCS	Glasgow Coma Scale
mRS	modified Rankin Scale
EVD	External ventricular drain
eGFR	Estimated glomerular filtration rate
aPTT	Activated partial thromboplastin time
MCV	Mean corpuscular volume
RDW	Red cell distribution width
MPV	Mean platelet volume
RLF	ReliefF
ANOVA	Analysis of Variance
Chi2	Chi-squared test
LIR	Linear Regression
LOR	Logistic Regression
CARTR	CART Regression
CARTC	CART Classification
RFR	Random Forest Regression
RFC	Random Forest Classification
Fisher	Fisher scale
RB	Gradient Boosting
NNs	Neural Networks
DTREEs	Decision Trees
SVMs	Support Vector Machines
KNNs	k-Nearest Neighbors
RF	Random Forest
LRC	Logistic Regression Classifier
GNB	Gaussian Naive Bayes
XGB	eXtreme Gradient Boosting
SGD	Stochastic Gradient Descent
BNB	Bernoulli Naive Bayes
EBTs	Ensemble Bagged Trees
ABC	AdaBoost
PPV	Positive predictive value
NPV	Negative predictive value
